# The Diagnosis and Treatment of Adenoids

**Published:** 1908-03-28

**Authors:** 


### Laryngology and Rhinology.

### THE DIAGNOSIS AND TREATMENT OF ADENOIDS.

The diagnosis of adenoids cannot be made with certainty from the facial appearance alone. The so-called "adenoid facies" is merely the appearance produced by nasal obstruction, and, while it is true that adenoid vegetations are by far the commonest cause of nasal obstruction in children, yet other forms of nasal inadequacy must be excluded before the diagnosis can be made. Enlargement of the turbinals and genuine hypertrophic rhinitis are not uncommon in children, and, though usually associated with adenoids, require to be treated if a satisfactory result is to be obtained. So also, septal deflections, hæmatomata, etc., occasionally occur even in quite young children, and, therefore, the nares must always be carefully examined before an operation for adenoids is undertaken. A rare cause of nasal obstruction in children is congenital malformation of the posterior nares or choanæ; one or both may be completely occluded by a bony partition, or the occlusion may be partial, leaving an opening of any size from the minutest orifice up to the normal. Much more common, and, therefore, more important, is a general smallness or insufficient growth of the cavity of the naso-pharynx, the roof of which, instead of being properly arched, stoops steeply to the posterior wall. In such cases any adenoid which is present should be thoroughly removed, but it is important to recognise the condition before operation, that the parents may be warned, for it is one of the causes of dissatisfaction after the operation. In older children, above the age of 10 or 12, the posterior ends of the inferior turbinals are often hypertrophied as the result of catarrh set up by the adenoids; if they have not been seen with the rhinoscope mirror they should be examined with the finger during the operation and, if enlarged, should be removed with a snare.

### Methods of Examination.

Whenever possible, adenoids should be examined for and diagnosed by posterior rhinoscopy, and this is usually possible in tractable children over the age of three or four. A very rapid examination is sufficient. The prominent posterior border of the septum is sought for and brought into view. This is like a slender column which, above, expands fan-wise into the concave vault of the naso-pharynx. If, however, a pad of adenoids be present, the concave expansion is interrupted by a hump or convexity, and, the larger the mass of adenoids, the lower does this hump descend, so that it may even hide the upper half or more of the septum. The presence of adenoids may thus be rapidly diagnosed at a glance, but it requires some practice to estimate the size of the mass and to determine whether it is large enough to indicate removal. From the position of the mirror the adenoid is seen much foreshortened, and appears smaller and less obstructive than is actually the case.

With nervous and frightened children such an examination is impossible, and it is better to proceed at once to digital exploration. The child is seated, the examiner stands at his right side and places the left arm round his head. The mouth is kept open by pushing in the left cheek between the teeth with the left hand; another excellent method is to retract the angle of the mouth with the left forefinger, and to introduce the tip of this finger between the jaws behind the molar teeth. It is impossible to get bitten in this way, and there is no risk of injury to the patient's cheek. The right forefinger is now passed rapidly to the right faucial pillar, along which it finds its way gently upwards into the naso-pharynx. It should not be passed up in the middle line, for contraction of the palate makes this difficult, and the pharynx is likely to be scratched. The soft and usually pleated feel of the adenoid can readily be recognised, and the finger should be removed instantly, for the patient's sensations, though not painful, are extremely unpleasant. It need hardly be said that the presence of blood on the examining finger is of no diagnostic value, but is a proof rather of the examiner's roughness than of the presence of adenoids.

Here again, though the presence of adenoids can be readily detected, the amount is more difficult to determine. Some quantity of lymphoid tissue is normal and anatomical on the naso-pharynx of children, and should only be removed if it causes symptoms. If these symptoms are only recent they may be due to a coryza, for a small amount of adenoid when inflamed becomes enlarged to a considerable size; in such cases the effect of a simple nasal lotion should be tried before resorting to operation.

### Indications for Removal.

When adenoid hypertrophy causes nasal obstruction, even if nocturnal, it should be removed, and this should be done early, before the age of seven or eight, for with the second dentition the jaws develop rapidly, and irremediable damage is often done by nasal obstruction at this period. On the other hand, the operation should not be performed in early infancy unless the obstruction be so great as to interfere with suckling, or unless there be suppurative otitis media.

The vegetations tend to shrivel about puberty, and, therefore, an adenoid which causes only slight obstruction in a patient approaching puberty may well be treated merely by nasal lotions. Sometimes, however, the growths remain large and obstructive far into adult life; more often the shrivelled and crenated mass harbours discharges, and keeps up a catarrhal inflammation, and for this reason has to be removed. Indeed, the influence of adenoids in causing and maintaining suppurative and catarrhal ear-disease is due rather to the catarrhal condition of the naso-pharynx than to any mechanical obstruction of the Eustachian tubes; from which it follows that aural discharge or deafness, even slight deafness noticeable only during a cold, is more important indication for the removal of adenoids than is nasal obstruction; it should be remembered that adenoids may be present and cause aural disease, although nasal respiration is perfectly free.

